# Association between the Dietary Inflammatory Index and Risk of Frailty in Older Individuals with Poor Nutritional Status

**DOI:** 10.3390/nu10101363

**Published:** 2018-09-23

**Authors:** Doyeon Kim, Yongsoon Park

**Affiliations:** Department of Food and Nutrition, Hanyang University, 222 Wangsimni-ro, Seongdong-gu, Seoul 04763, Korea; kdy2313@hanyang.ac.kr

**Keywords:** dietary inflammatory index, nutritional status, frailty, older individuals

## Abstract

Both inflammation and poor nutritional status are major risk factors of frailty, and the dietary inflammatory index (DII) has been suggested as being associated with the risk of frailty. The present study aimed to investigate whether DII scores were positively associated with the risk of frailty in older individuals, particularly those with poor nutritional status. In total, 321 community-dwelling older individuals aged 70–85 years were recruited and categorized as non-frail, pre-frail, and frail according to the Cardiovascular Health Study index. DII scores were calculated based on 24-h dietary recall, and nutritional status was assessed using the Mini Nutritional Assessment. Multinomial logistic regression analysis showed that DII scores were positively associated with the risk of frailty in older individuals (odds ratio, OR 1.64, 95% confidence interval, 95% CI 1.25–2.17), particularly those with poor nutritional status (OR 1.68, 95% CI 1.21–2.34). Among the frailty criteria, weight loss (OR 1.29, 95% CI 1.03–1.60), low walking speed (OR 1.33, 95% CI 1.10–1.61), and low grip strength (OR 1.34, 95% CI 1.13–1.60) were associated with DII scores. In addition, the optimal DII cut-off score for frailty was ≥0.93 (sensitivity 71%; specificity: 72%; area under the receiver operating characteristic curve, AUC = 0.792). The present study showed that a pro-inflammatory diet was associated with increased risk of frailty, particularly in older individuals with poor nutritional status. Future randomized controlled trials with a low DII diet for the prevention of frailty are needed to confirm our finding.

## 1. Introduction

Frailty is characterized by slowness, weakness, exhaustion, low physical activity, and unintentional weight loss, and this condition is associated with a high risk for subsequent falls, hospitalization, disability, and mortality [1]. Korea is one of the most rapidly aging countries worldwide [2], with a 13% prevalence of frailty among Korean elderly over 65 years of age [3]. 

One of the major risk factors of frailty is malnutrition, which can accelerate the age-associated decline in muscle mass and strength [4]. Malnutrition is commonly assessed using the Mini Nutritional Assessment (MNA) in older individuals, and MNA questions and frailty criteria overlap, particularly those related to physical weakness and weight loss [5]. A previous study has reported that approximately 90% of older individuals at risk of malnutrition based on the MNA were frail or pre-frail [6]. In addition, malnutrition interacts with inflammation in a vicious cycle whereby it not only increases the risk and severity of inflammation, but is also a result of inflammation [7].

Although the pathophysiological changes underlying and preceding frailty are unclear, inflammation may be significantly associated with frailty [8]. A meta-analysis showed that pro-inflammatory cytokines, particularly c-reactive protein (CRP) and interleukin-6 (IL-6), were associated with the risk of both pre-frailty and frailty [9]. In addition, reactive oxygen species (ROS) have been shown to stimulate receptor activator nuclear factor-κB (NF-κB) ligand and tumor necrosis factor-alpha (TNF-α), may participate in normal aging processes, and have an effect on the onset and progression of age-related diseases [10].

The dietary inflammatory index (DII) is a useful dietary tool developed to measure the inflammatory potential of diet [11], and it is associated with inflammatory markers, including CRP, IL-6, and TNF-α [12,13,14]. Previous epidemiologic studies reported that DII scores were associated with glucose intolerance [15], asthma [16], bone mineral density of the hip [17], breast cancer [18], and colorectal cancer [19]. Shivappa et al. [20] reported that DII scores were positively associated with the incidence of frailty assessed using the Study of Osteoporotic Fractures (SOF) instrument in participants with or at risk of knee osteoarthritis during a follow-up period of 8 years. However, no study that investigated the association between DII scores and the risk of frailty in older individuals, particularly those with poor nutritional status, has been conducted. Thus, the present study aimed to examine whether DII scores are positively associated with the risk of frailty in older individuals, and the association was compared based on nutritional status.

## 2. Materials and Methods 

### 2.1. Patient Population

In total, 357 community-dwelling older individuals aged 70–85 years were recruited consecutively at four welfare centers in Seoul, Korea, from 2016 to 2017. Thirty-six older individuals were excluded due to missing data on dietary intake, age, and MNA score. Data, including age, sex, medical history, chewing ability, and dietary intake were obtained from the older individuals by trained interviewers. This study was conducted according to the guidelines in the Declaration of Helsinki, and all procedures were approved by the institutional review board of Hanyang University (HYI-15-228). Written informed consent was obtained from the participants.

### 2.2. Frailty Assessment

Frailty was diagnosed following the Cardiovascular Health Study (CHS) frailty index based on five criteria [1]: weight loss, exhaustion, low physical activity, low walking speed, and low handgrip strength. Weight loss was defined as self-reported unintentional weight loss ≥ 4.5 kg within the previous year. Exhaustion was evaluated using the Center for Epidemiological Studies Depression scale, and low physical activity was calculated as the energy spent for a week using the International Physical Activity Questionnaire. Low walking speed was defined as ≤0.8 m/sec from the average of the walking speed repeated three times for 4 m with 1.5 m before and after the walkway to allow for acceleration and deceleration. Low handgrip strength of both hands was measured twice at standing position with the arms outstretched in a 30° angle using a hand dynamometer (Takei, Niigat, Japan) by the same operator for all participants. Average of grip strength in the dominant hand was used after adjusting for gender and body mass index (BMI). Older individuals who met three or more criteria were considered frail, those who met one or two criteria were regarded pre-frail, and those who did not meet any of the criteria were considered non-frail.

### 2.3. Mini Nutritional Assessment (MNA)

The MNA consists of anthropometric measurements, general assessments, dietary questionnaires, and self-assessments [21]. Body weight was measured with an electronic scale (BioSpace, Chungcheong-do, Korea) to the nearest 0.1 kg, and height was measured with an extensometer (Samhwa, Incheon, Korea) to the nearest 0.1 cm. Using a nonelastic tapeline, the mid-upper arm circumference was measured on the nondominant relaxed arm midway between the tip of the acromion and olecranon process, and calf circumference was measured on the nondominant, thickest part of the undressed calf. Based on the final score, older individuals were categorized into three groups: >24, with good nutritional status; 17–23.5, at risk of malnutrition; and <17, with malnutrition. Poor nutritional status included both the risk of malnutrition and malnutrition.

### 2.4. Dietary Inflammatory Index (DII)

Dietary intake was assessed by a registered dietitian using the 24-h dietary recall and was analyzed using the CAN-Pro version 4.0 (Computer Aided Nutritional analysis program, Korean Nutrition Society, Korea). The data obtained using the 24-h dietary recall were used to calculate DII scores, which consist of 45 food parameters, including macro- and micronutrients, flavonoids, spices, and food items, each associated with an inflammatory effect score [11]. The DII scores obtained in this study included data on 33 of the 45 possible parameters comprising the DII: carbohydrate, fat, protein, fiber, vitamin A, vitamin D, vitamin E, vitamin C, beta carotene, thiamin, riboflavin, niacin, vitamin B_6_, folate, vitamin B_12_, magnesium, iron, zinc, selenium, *n*-3 fatty acids, *n*-6 fatty acids, cholesterol, saturated fat, polyunsaturated fatty acids, monounsaturated fatty acids, garlic, ginger, onion, turmeric, green/black tea, pepper, alcohol, and caffeine. Energy, one of the parameters of the DII, was adjusted for the analyses. Eleven food parameters including eugenol, rosemary, saffron, thyme/oregano, flavan-3-ol, flavones, flavonols, flavonones, anthocyanidins, isoflavones, and trans fat, were not included in the present study because they were not available from the database. Thirty-three possible parameters were used to calculate the participant’s exposure relative to the standard global mean as a z-score, and this value was divided by the parameter’s standard deviation. To minimize the effect of right skewing, this value was converted to a centered percentile score by doubling and subtracting 1. The derived value was then multiplied by the respective food parameter effect score, which was obtained from a literature review of 1943 articles that were scored [11]. The participant’s DII score was computed by multiplying this value by the specific DII score for each food parameter and by summing together all these 33 values according to the following formula: DII = b_1_ × n_1_ + b_2_ × n_2_ + ……….. + b_33_ × n_33_, where b*_i_* refers to the literature-derived inflammatory effect score for each of the evaluated food parameters and n*_i_* refers to the food parameter-specific centered percentiles, which were derived from the dietary data, for each *i* from 1 to 33 [11]. Table 1 shows the inflammatory effect scores for dietary components used for calculation of DII. Theoretical DII scores ranged from –9 (maximally anti-inflammatory diet) to +8 (maximally pro-inflammatory diet). 

### 2.5. Statistical Analyses

The Statistical Package for the Social Sciences software version 24.0 (SPSS Inc., Chicago, IL, USA) was used for statistical analyses, and a *p*-value < 0.05 was considered statistically significant. Continuous variables were expressed as mean ± standard deviation (SD), and differences were verified with Bonferroni’s post hoc test after using one-way analysis of variance (ANOVA). The proportions of nominal variables were presented as the number of participants (percentage distribution) using the chi-squared test. 

Correlations between frailty score and the risk factors of frailty were calculated using the Spearman’s rank correlation coefficients. Associations between the DII score and the risk of frailty or pre-frailty vs. non-frailty were assessed using multinomial logistic regression adjusted for age, chewing ability, and energy intake. The multivariable logistic regression was used to evaluate the associations between the DII score and the risk of each frailty criterion adjusted for age, chewing ability, and energy intake.

Analysis of the receiver operating characteristic (ROC) curve was conducted to determine the optimal cut-off values of DII for frailty with the maximum Youden index (sensitivity + 1-specificity). In addition, the areas under the ROC curve (AUC) were used to determine the reliability as predictive markers of frailty. The AUC values indicate the predictive ability of the model: >0.9, very good; >0.8, good; and >0.7, useful [22].

## 3. Results

### 3.1. Characteristics of Participants

Participants were categorized as frail (*n* = 42), pre-frail (*n* = 187), and non-frail (*n* = 92) (Table 2). The frail older individuals were significantly older than non-frail older individuals, and the DII score of the frail older individuals was higher than that of the pre-fail and non-frail older individuals. Energy intake and MNA score were lowest in the frail older individuals, and poor chewing ability and malnutrition were more common in the frail older individuals. However, no significant differences were observed in terms of sex, BMI, and medical history among the three groups.

### 3.2. Association between DII and Frailty Status

Frailty score was positively associated with DII score and age but not with energy intake in older individuals (Table 3). A significant association was observed between frailty score and the risk factors of frailty in older individuals with poor nutritional status but not in those with good nutritional status.

In the multinomial regression analysis that used non-frail as the reference group, the DII score was positively associated with the risk of frailty after adjusting for age, chewing ability, and energy intake (Table 4). In particular, the DII score was associated with the risk of frailty in older individuals with poor nutritional status but not in those with good nutritional status.

Multivariate-adjusted logistic regression analysis showed that the DII score was positively associated with the risk of weight loss, low walking speed, and low grip strength after adjusting for confounding variables (Table 5). In addition, the DII score was associated with the risk of low walking speed and low grip strength in older individuals with poor nutritional status but not in those with good nutritional status.

### 3.3. Predictability of Frailty Using the DII

**Figure 1:**
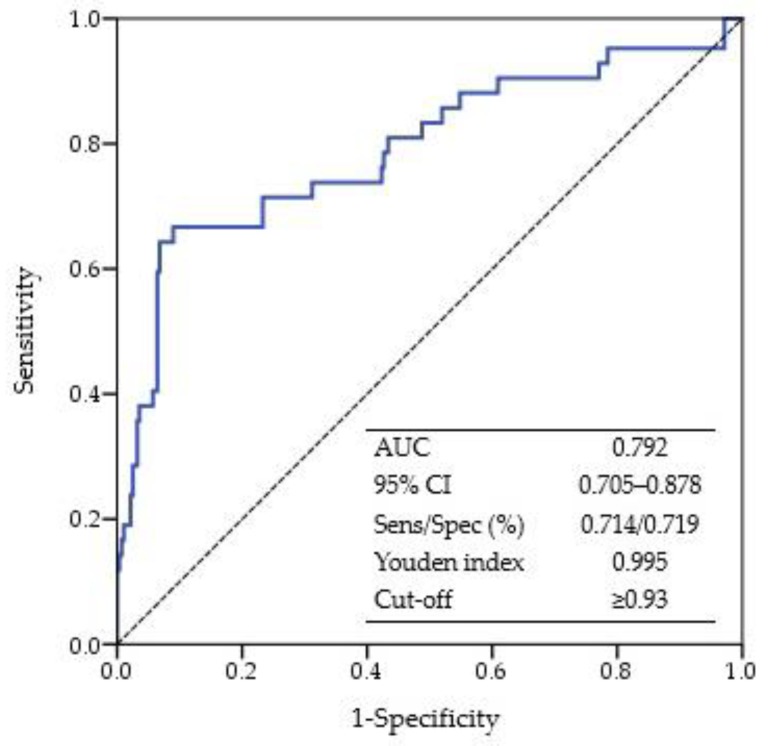
ROC curve for the dietary inflammatory index score in predicting frailty. ROC curve, receiver operating characteristic curve; AUC, area under the ROC curve; CI, confidence interval; Sens, sensitivity; Spec, specificity.

The performance of the DII score was useful in discriminating frailty among older individuals, given an AUC of 0.792 (Figure 1). The best cut-off value for the DII score was ≥0.93, with the largest Youden index at 0.995 to determine frailty among older individuals.

## 4. Discussion

The present study showed that the DII scores, indicating inflammatory diet, were positively associated with the risk of frailty in older individuals, particularly in those with poor nutritional status. In addition, the DII scores were positively associated with the risk of weight loss, low walking speed, and low grip strength based on the frailty criterion. 

The previous study reported that the DII scores were positively associated with the risk of frailty assessed using the SOF index in participants with or at risk of knee osteoarthritis [20]. Unlike the result of the present study, that of the previous study [20] might not be generalizable to older individuals since only participants with or at risk of knee osteoarthritis were included. In the present study, frailty was assessed using the CHS index since Jung et al. [3] reported that the CHS index but not the SOF index was positively associated with mortality, functional decline, and hospitalization in older individuals. Consistent with the present study, previous studies reported that prevalence of frailty increased with aging, and older individuals with frailty were older than non-frail individuals [1,6,23].

Higher DII scores have been shown to associate with inflammatory cytokines, including CRP [12,24,25], IL-6 [13,26], and TNF-α [14]. A meta-analysis of 32 cross-sectional studies has reported that higher levels of inflammatory cytokines, such as CRP and IL-6, were associated with the risk of frailty in participants with a mean age of 75 years [9]. The relationship between inflammation and frailty was more significant in older individuals with advancing age. Both inflammatory levels and frailty were positively associated with negative outcomes, such as chronic diseases, hospitalization, and mortality [27,28], indicating that common pathways were responsible for inflammation and frailty. Moreover, NF-κB is rapidly activated by TNF-α, and TNF-α/NF-κB signaling is regulated by endogenous ROS in the mouse-derived C2C12 muscle cells and the primary cultures from rat skeletal muscles, suggesting that TNF-α directly induces skeletal muscle protein loss [29].

Sarcopenia, defined as an abnormal loss of muscle mass and strength, is a common symptom in the frail older individuals [30]. A meta-analysis of 17 cross-sectional studies has reported that inflammatory cytokines, particularly CRP, were positively associated with the risk of sarcopenia in participants with a mean age of 66 years [31]. In addition, the levels of CRP and IL-6 were positively associated with the risk of low handgrip strength in the Longitudinal Aging Study Amsterdam [32]. Taaffe et al. [33] also showed that the levels of CRP and IL-6 were negatively associated with walking speed and hand grip strength in older individuals. In the Woman’s Health and Aging Study, older women with higher levels of IL-6 experienced a steeper decline in walking speed than those with lower levels of IL-6 [34]. 

Unintentional weight loss was also associated with the risk of frailty [1] and mortality [35] in older individuals. In the Iowa Women’s Health Study, one or more episodes of unintentional weight loss of more than 20 pounds was associated with a higher mortality rate in women aged 55–69 years [36]. In addition, unintentional weight loss but not intentional weight loss was associated with the risk of higher all-cause mortality in older men after adjusted for all chronic diseases [37]. Ruscin et al. [38] showed that in cases of unintentional weight loss there were increased circulating levels of TNF-α in older individuals. On the other hand, weight loss, especially if involuntary, is not a normal part of aging and may represent some underlying disease process [4]. Cytokines such as TNF-α and IL-6 have been shown to suppress the appetite and promote muscle and fat breakdown, suggesting that pro-inflammatory cytokines may induce involuntary weight loss [39].

The other major findings of this study showed that higher DII scores were associated with the risk of frailty in older individuals with poor nutritional status but not in those with good nutritional status. Malnutrition, which is prevalent in the geriatric population, is one of the main risk factors of the onset of frailty [40]. Nutritional frailty refers to a disability in older individuals owing to rapid, unintentional loss of body weight and lean body mass [4]. In the Chianti cohort study, a daily energy intake ≤21 kcal/kg was significantly associated with the risk of frailty among older individuals [41]. In addition, caloric intake was positively associated with chewing ability in older individuals [42], which was more common in frail than pre-frail or non-frail older individuals [43]. The present study also demonstrated that frail older individuals had lower energy intake and poorer chewing ability than non-frail individuals. Kim et al. [44] reported that the levels of IL-6 and TNF-α were negatively associated with nutrient adequacy ratios as well as physical performance assessed using the short physical performance battery in the frail older individuals. In addition, frail older patients who were hospitalized had lower levels of albumin, which is a marker of malnutrition, and higher levels of CRP and IL-6 than non-frail individuals [45]. Biomarkers for malnutrition (albumin and transthyretin) and inflammation (CRP and α1-acid glycoprotein) were significantly associated with mortality in older men [46].

The present study has several limitations. First, the relatively small number of frail older individuals might have attenuated the strength of or underestimated our results. Second, 11 of 45 food parameters were not available for the DII calculation in the present study. Shivappa et al. [11] reported that dropping from the maximum 45 to 28 parameters did not result in a decrease in the predictive capability of the DII, indicating that missing food parameters would not have a major impact on the scoring in the present study. Third, trans fat, a pro-inflammatory parameter, has been known to associate with DII [11], but was not included in the present study because the database of CAN-PRO 4.0 did not include trans fat. Fortunately, intake of trans fat was only 0.36 g/d (0.12% energy) in Korean aged 5–70 years and was lower in Korean aged 20–70 years, 0.18g/d (0.06% energy), suggesting that trans fat intake was very low and decreased with increasing age in Koreans [47]. Therefore, intake of trans fat may be too low to influence DII in the Korean population. Fourth, dietary intake was measured by one day of 24-h recall, which might not be reflective of the participants’ long-term dietary intake. However, the use of Food-frequency Questionnaire may be limited in elderly population due to poor memory [48]. Sun et al. [49] demonstrated that one day of 24-h recall was a reliable and predictive method for older individuals based on weight changed during 6-month follow-up. Fifth, although adjustments were made for confounders, unmeasured factors might have affected the results of this study. Finally, because of the cross-sectional study design, this study was not able to identify the causal relationship between DII and frailty in older individuals.

## 5. Conclusions

This study is the first to show that DII scores are positively associated with the risk of frailty in older individuals with poor nutritional status. In addition, among the frailty criteria, weight loss, low walking speed, and low grip strength were positively associated with higher DII scores. However, to our knowledge, there are no intervention trials. Thus, future studies must be conducted to investigate whether dietary interventions focused on diets rich in anti-inflammatory compounds could reduce the risk of frailty based on nutritional status.

## Figures and Tables

**Table 1:** Inflammatory effect scores for dietary components used for calculation of the dietary inflammatory index (DII).

Dietary Parameters	Inflammatory Effect Score ^1^
Carbohydrate (g)	0.097
Total fat (g)	0.298
Protein (g)	0.021
Fiber (g)	−0.663
Vitamin A (μg RE)	−0.401
Vitamin D (μg)	−0.446
Vitamin E (mg)	−0.419
Vitamin C (mg)	−0.424
Beta carotene (μg)	−0.584
Thiamin (mg)	−0.098
Riboflavin (mg)	−0.068
Niacin (mg)	−0.246
Vitamin B_6_ (mg)	−0.365
Folate (μg)	−0.190
Vitamin B_12_ (mg)	0.106
Magnesium (mg)	−0.484
Iron (mg)	0.032
Zinc (mg)	−0.313
Selenium (μg)	−0.191
*n*-3 fatty acids (g)	−0.436
*n*-6 fatty acids (g)	−0.159
Cholesterol (mg)	0.110
Saturated fat (g)	0.373
Polyunsaturated fatty acids (g)	−0.337
Monounsaturated fatty acids (g)	−0.009
Garlic (g)	−0.412
Ginger (g)	−0.453
Onion (g)	−0.301
Turmeric (mg)	−0.785
Green/black tea (g)	−0.536
Pepper (g)	−0.131
Alcohol (g)	−0.278
Caffeine (g)	−0.110

^1^ A negative value indicates anti-inflammatory effect and a positive score indicates pro-inflammatory effects. RE, retinol equivalents.

**Table 2:** Characteristics of older individuals according to the three frailty categories.

Variables	Non-Frail (*n* = 92)	Pre-Frail (*n* = 187)	Frail (*n* = 42)	*p*-Value
Age (years)	75.98 ± 3.77 ^a^	76.78 ± 3.73 ^ab^	77.88 ± 3.79 ^b^	0.023
Female, *n* (%)	60 (65.2)	129 (69.0)	29 (69.0)	0.087
BMI (kg/m^2^)	24.66 ± 3.34	24.68 ± 3.26	24.45 ± 3.36	0.918
Medical history, *n* (%)				
Diabetes	18 (19.6)	51 (27.3)	15 (35.7)	0.124
Cardio-cerebrovascular disease	58 (63.0)	140 (74.9)	31 (73.8)	0.113
Gastrointestinal disease	4 (4.3)	16 (8.6)	0 (0.0)	0.079
Musculoskeletal disease	25 (27.2)	45 (24.1)	14 (33.3)	0.451
Depression	1 (1.1)	7 (3.7)	1 (2.4)	0.443
Chewing ability, *n* (%)				
Poor	20 (21.7)	71 (38.0)	3 (64.3)	<0.001
Moderate	9 (9.8)	17 (9.1)	2 (4.8)	
Good	63 (68.5)	99 (52.9)	13 (31.0)	
Energy intake (kcal)	1438.6 ± 414.3 ^a^	1305.2 ± 362.1 ^b^	1055.5 ± 340.9 ^c^	<0.001
MNA score	24.09 ± 2.84 ^a^	22.03 ± 3.01 ^b^	19.76 ± 2.79 ^c^	<0.001
MNA category, *n* (%)				
Well-nourished	53 (57.6)	47 (25.1)	3 (7.1)	<0.001
At risk of malnutrition	38 (41.3)	134 (71.7)	33 (78.6)	
Malnutrition	1 (1.1)	6 (3.2)	6 (14.3)	
DII score	−0.81 ± 2.06 ^a^	−0.15 ± 2.18 ^a^	2.27 ± 2.53 ^b^	<0.001

Data were presented as mean ± SD number of the participants (percentage distribution), as appropriate; Values with different superscript letters in the same row were significantly different among the three groups according to ANOVA with Bonferroni’s post hoc test; BMI, body mass index; MNA, mini nutritional assessment; DII, dietary inflammation index.

**Table 3:** Correlation between frailty score and the risk factors of frailty according to nutritional status.

Variables	Correlation Index	Total (*n* = 321)	Good Nutritional Status (*n* = 103)	Poor Nutritional Status (*n* = 218)
DII score	Spearman’s ρ	0.369 **	0.162	0.352 **
	*p*-value	<0.001	0.103	<0.001
Age	Spearman’s ρ	0.170 **	0.142	0.143 *
	*p*-value	0.002	0.151	0.035
Energy intake	Spearman’s ρ	−0.289 **	−0.144	−0.251 **
	*p*-value	<0.001	0.146	<0.001

** Correlation is significant at the 0.01 level; * Correlation is significant at the 0.05 level; DII, dietary inflammatory index.

**Table 4:** Association between the dietary inflammatory index score and the risk of pre-frailty and frailty according to nutritional status.

	Total (*n* = 321)	Good Nutritional Status (*n* = 103)	Poor Nutritional Status (*n* = 218)
Non-frail/pre-frail, *n*	92/187	53/47	39/140
Crude OR (95% CI)	1.15 (1.02–1.29) *	1.01 (0.83–1.23)	1.11 (0.94–1.30)
Adjusted OR (95% CI) ^1^	1.01 (0.85–1.19)	0.86 (0.65–1.14)	1.10 (0.87–1.39)
Non-frail/frail, *n*	92/42	53/3	39/39
Crude OR (95% CI)	1.89 (1.55–2.31) **	2.15 (1.16–3.97) *	1.73 (1.38–2.19) **
Adjusted OR (95% CI) ^1^	1.64 (1.25–2.17) **	3.13 (0.96–10.20)	1.68 (1.21–2.34) **

^1^ Adjusted for age, chewing ability, and energy intake; OR, odds ratio; CI, confidence interval. * *p*-value < 0.05; ** *p*-value < 0.01.

**Table 5:** Association between the dietary inflammatory index score and the risk of each frailty criterion according to nutritional status.

	Total (*n* = 321)	Good Nutritional Status (*n* = 103)	Poor Nutritional Status (*n* = 218)
Weight loss/non-weight loss, *n*	38/283	4/99	34/184
Crude OR (95% CI)	1.15 (1.02–1.29) *	1.26 (0.82–1.94)	1.09 (0.93–1.28)
Adjusted OR (95% CI) ^1^	1.29 (1.03–1.60) *	1.50 (0.82–2.74)	1.26 (0.99–1.60)
Exhaustion/non-exhaustion, *n*	184/137	31/72	153/65
Crude OR (95% CI)	1.16 (1.05–1.27) **	0.96 (0.78–1.17)	1.11 (0.98–1.26)
Adjusted OR (95% CI) ^1^	1.04 (0.90–1.20)	0.89 (0.67–1.19)	1.08 (0.89–1.30)
Low physical activity/non-low physical activity, *n*	43/278	6/97	37/181
Crude OR (95% CI)	1.41 (1.21–1.63) **	1.50 (1.04–2.18) *	1.33 (1.13–1.58) **
Adjusted OR (95% CI) ^1^	1.16 (0.94–1.45)	1.52 (0.90–2.57)	1.08 (0.85–1.37)
Low walking speed/non-low walking speed, *n*	65/256	11/92	54/164
Crude OR (95% CI)	1.43 (1.25–1.62) **	1.45 (1.08–1.95) *	1.37 (1.18–1.59) **
Adjusted OR (95% CI) ^1^	1.33 (1.10–1.61) **	1.35 (0.90–2.02)	1.33 (1.07–1.65) *
Low grip strength/non-low grip strength, *n*	74/247	22/81	52/166
Crude OR (95% CI)	1.28 (1.14–1.44) **	1.25 (1.00–1.55)	1.33 (1.15–1.54) **
Adjusted OR (95% CI) ^1^	1.34 (1.13–1.60) **	1.28 (0.94–1.74)	1.37 (1.11–1.70) **

^1^ Adjusted for age, chewing ability, and energy intake; OR, odds ratio; CI, confidence interval. * *p*-value < 0.05; ** *p*-value < 0.01.
